# The Relative Contributions of NIH and Private Sector Funding to the Approval of New Biopharmaceuticals

**DOI:** 10.1007/s43441-022-00451-8

**Published:** 2022-09-03

**Authors:** Duane Schulthess, Harry P. Bowen, Robert Popovian, Daniel Gassull, Augustine Zhang, Joe Hammang

**Affiliations:** 1 Vital Transformation, Wezembeek-Oppem, Belgium; 2 McColl School of Business, Queens University of Charlotte, Charlotte, NC USA; 3 Global Healthy Living Foundation & Senior Health Policy Fellow Progressive Policy Institute, Washington, DC USA; 4 University of Southern California, Los Angeles, CA USA

**Keywords:** NIH funding, FDA approval, March-in Rights, Bayh–Dole Act

## Abstract

**Objectives:**

There remains ongoing debate regarding the relative efficacy of public (NIH) and private sector funding in bringing biopharmaceutical innovations to market. This paper investigates the significance of each party’s level of funding for obtaining Food and Drug Administration (FDA) authorization.

**Methods:**

A cohort of research projects linked to 23,230 National Institute of Health grants awarded in the year 2000 was audited to account for patents, where the project led to a product in clinical development and potentially FDA approval. A total of 8126 associated patents led to the identification of 41 therapies that registered clinical trials; 18 of these therapies received FDA approved.

**Results:**

NIH funding for the 18 FDA-approved therapies totaled $0.670 billion, whereas private sector funding (excluding post-approval funding) totaled $44.3 billion. A logistic regression relating the levels of public and private funding to the probability of FDA approval indicates a positive and significant relationship between private sector funding and the likelihood of FDA approval (*p* ≤ 0.0004). The relationship between public funding and the likelihood of FDA approval is found to be negative and not statistically significant.

**Conclusion:**

Our study results underscore that the development of basic discoveries requires substantial additional investments, partnerships, and the shouldering of financial risk by the private sector if therapies are to materialize as FDA-approved medicine. Our finding of a potentially negative relationship between public funding and the likelihood that a therapy receives FDA approval requires additional study.

## Introduction

There is a consensus that the public and private sectors play complementary roles in the discovery and development of new therapies (e.g., see [1]). Both segments conduct basic, translational, and clinical research; however, each sector's focus and sources of funding are different. The federal government is the primary funder of basic research in biomedical sciences through the National Institutes of Health (NIH). This research is essential for informing all medical progress, including the development of therapies. Overall, 54% of basic science milestones are achieved by the public sector and 27% by the private sector [2]. From that point onward, taking the necessary risks associated with the drug development process required to advance basic science research into safe and effective treatments for patients corresponds primarily to the biopharmaceutical industry. Performing Phase I through IV clinical trials consumes more than 90% of total research and development (R&D) cost [3].

A number of recent studies indicate that a majority of this R&D is funded by investments made by the private sector.^1^ In a 2019 report, Research America indicated that, in 2016, the private sector funded 67% of total U.S. medical and health R&D while the federal government supported 22% [4]. The organization also reported that, in 2018, the biopharmaceutical industry invested $102 billion in R&D, whereas the entire NIH budget for that year was $35.4 billion [4]. IQVIA’s Institute for Human Data Science reported in May of 2021 that "aggregate R&D expenditures by the 15 companies with the highest pharmaceutical sales reached $123 billion in 2020 and exceeded 20% of sales for the first time, while venture capital flows into the life sciences rose by 50% in 2020 over 2019" and composite success rate among all therapeutic areas reached 9.8% [5]. A 2019 study found that 75% of all U.S. Food and Drug Administration (FDA) approved drugs—excluding new vaccines, biologic medicines, and gene therapies—between January 2008 and December 2017 were fully funded and researched by private companies [6]. The results were comparable with those of an earlier study that found only 9% of new drugs approved between 1988 and 2005 had either a government interest statement disclosure or a government agency first-listed as a patent assignee [7]. Finally, research published in 2011 by Rohrbaugh et al. found that the Bayh–Dole Act (Public Law 96–517) had been responsible for the transfer of federally funded research and intellectual property that had led directly to 153 FDA-approved drugs that were discovered, at least in part, by public-sector research from a total of 1541 approved therapies [8], less than 10% of the total.

Despite a plethora of evidence regarding the relative importance of private versus public (NIH) funding, there is a common perception that, in the U.S., public funding is the primary engine responsible for the emergence of new and innovative therapies with the private sector simply cherry-picking winning biopharmaceutical assets. Recently, a highly quoted study from Bentley University made the claim that the COVID-19 treatment drug remdesivir had received, “$6.5 billion in NIH funding,” and further stated that this, “underscores the scale and significance of the public-sector investments that enable new drug discovery and development.” [9]. However, a Government Accountability Office (GAO) report investigated this claim and concluded, “Gilead’s collaborations with government scientists with respect to remdesivir generated no intellectual property rights for federally funded researchers or government agencies.” [10].

While the total amount of funds dedicated to commercial R&D are overwhelmingly driven by the private sector, studies analyzing the relative importance of private vs public funding are often based upon the retrospective analysis of therapies that have been successfully approved by the FDA. Working backwards from the point of FDA approval biases the study cohort and neglects the totality of the financial contributions from the point of initial research and IP creation, through to the suspension of clinical development or marketing authorization.

The question of whether private funding is statistically significant to the probability of a given therapy’s FDA approval has been a core focus of our research. Our 2019 publication in Therapeutic Innovation and Regulatory Science found that, “The amount invested in a company was statistically significant to successfully bringing a product to market P < 0.0002.” [11]. Further, our 2020 publication found that the total sum of private investments made before FDA approval are also a very good predictor of a therapy’s future revenues. It states, “The relationship between investments and future annual revenues is statistically significant (p < 0.0001). As well, the estimated relationship explained by the regression between investments and revenues accounts for 77% of the model’s variability (R^2^ = 0.773)” [12]. However, this still leaves as an open question the relative importance of both public and private sector funding on FDA approvals.

In a 2010 study of the San Diego biotechnology cluster, Radu Munteanu investigated this relationship by analyzing the likelihood of a biotech firm’s success as measured by several intrinsic characteristics [13]. The study found that, “The success of firms is positively affected by the [initial public offering] IPO amount.” Munteanu also states that spinning out a biotech firm from the University of California San Diego (UCSD) “affects negatively the probability of success...a more detailed investigation of this variable would be necessary before a clear interpretation can be provided.” During the period of Munteanu’s analysis, the San Diego region received $3.2b in NIH grants [13]. This leads to the following, perhaps uncomfortable, hypothesis; higher levels of NIH funding negatively impact a given biotech firm’s chance of success.

In this paper, we test and expand upon this hypothesis by first mapping NIH grants in a single year (2000) to therapies that entered clinical trials, with some receiving FDA approval. We then examine for the impact and statistical significance of the levels of NIH and private funding for the probability of FDA approval.

## Research Questions

The research addresses three main questions. First, how many NIH research grants from a single year contribute to patented discoveries associated with an FDA-approved medicine? Second, in cases where patents linked to NIH-funded research are associated with therapies in development, what are the relative financial contributions of the NIH and the private sector to the development of those therapies? Finally, are public and private funding statistically significant predictors of the probability of FDA approval and, if so, what is the absolute and relative impact of each source of funding to the likelihood of FDA approval?

The findings of this study are intended to add to the body of evidence on the relative contributions of the public and private sectors in bringing new biopharmaceuticals to the US market. Clarifying this issue is directly relevant to current regulatory debates between the U.S. House, Senate, and Biden Administration over Federal March-in Rights, the right for the US Government to reclaim IP that was funded by the public and licensed to a private firm, “if the Federal agency determines that such action is necessary to alleviate health or safety needs which are not reasonably satisfied…” [14].

## Data Methods and Sources

To identify a cohort of therapies that received NIH funding, and the total amount of that funding, we first identified 23,230 NIH extramural grants in the year 2000 across six major NIH institutes and centers.^2^ Using data from the NIH Research Portfolio Online Reporting Tools (RePORT), we then identified 8126 patents linked to discoveries funded by these NIH grants. These 8126 patents were then linked to data from BioMedTracker and ClinicalTrials.gov to identify all therapies associated with the NIH-funded patents that reached clinical trials through 2021. This resulted in the identification of a cohort of 41 therapies associated with 135 NIH derived patents that underwent clinical trials, of which 18 were approved by the FDA.

**Figure 1.**
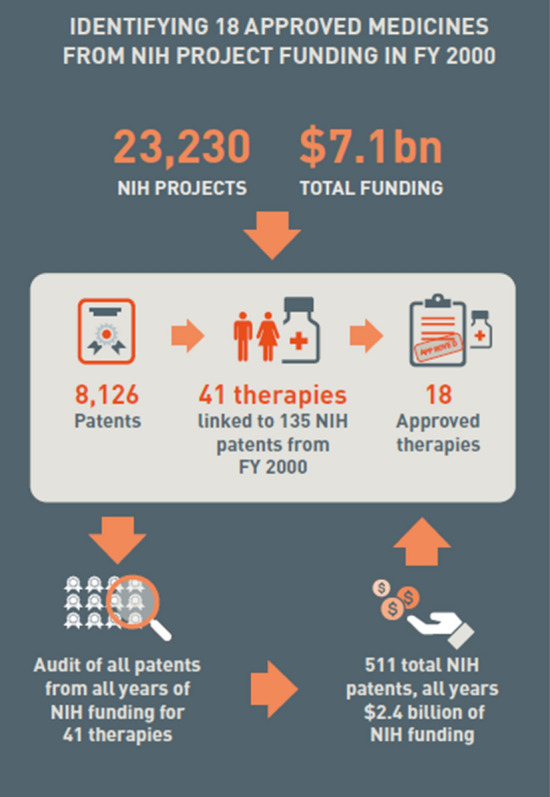
Search Strategy for all NIH Grants in All Years Linked to Each of Forty-One Therapies Initially Identified as Having Patents Derived from NIH Grants Issued in Year 2000.

As illustrated in Fig. 1, an additional manual search was then conducted for all years of NIH funding included in the NIH RePORT database for all registered patents associated with our 41 therapies. This search yielded an additional 376 NIH-funded patents across all years. Hence, our cohort of 41 therapies was associated with 511 total patents derived from 1181 NIH grants running multiple years from 1984 through 2021. This additional search means that, even if a grant was extended well beyond the awarding of a patent included in the portfolio of any one of our 41 therapies, we still included the entire grant amount over all award years when computing the total amount of NIH funding for a given therapy. While this extensive multi-year grant search for additional funding likely overstates the total financial contributions of the NIH, this was done to err on the side of caution and avoid any potential criticisms of under-counting the public contributions toward any of our 41 therapies,

Data on private sector funding before and after 2000 for each of our 41 therapies were derived from Securities and Exchange Commission 10-K audits, corporate reports, BioMedTracker, and BioCentury’s BCIQ database, including funding Activity Codes, P01 Program Projects, R01 Equivalents, DP2, R01, RF1, SBIR/STTR, R41, R42, R43, R44. These types of funding encompass equity, royalty, licensing, IPO, acquisition, debt, and finance transactions.

Finally, the cumulative totals of public and private funding for each of the 41 identified therapies was computed by summing the respective US dollar amounts, unadjusted for inflation, at each point in time. For therapies that received FDA approval, our measure of total private funding excludes any amounts that occurred after the approval date.

## Analysis and Results

Table 1 shows the data on public (NIH) and private funding levels for each of the 41 investigational therapies identified by our research. For these 41 therapies, NIH funding totaled $2415 billion while pre-FDA approval private sector funding totaled $50,671 billion (≈ 95.5% of all funding). If post-FDA approval funding is also included, total private funding rises to $91,256 billion ((≈ 97.4% of all funding). Table 1 also shows the year in which 18 of our 41 therapies in our cohort received FDA approval. As shown in the last line of Table 1, NIH funding for those 18 therapies totals $670 million, while private sector investment—which exceeded NIH funding for 17 of them—totaled $44.3 billion (≈ 98.5% of all funding). Oncolytic drugs accounted for the largest number (5) of approved therapies among the 18. The average private funding per cancer therapy was $5.5 billion, the average public contribution per cancer therapy was $5 million.

**Table 1:** Total Public (NIH) and Private Funding for Cohort of Forty-One Therapies.

Therapy	Total Public Funding ($ Mil)	Total Private Funding^a^ ($ Mil)	Year Approved
IMMU-132 /(Trodelvy)	$0.850	$22,519.457	2020
Tysabri	$7.575	$8756.691	2004
Myalept	$8.332	$3179.600	2014
Nexavar	$5.305	$1384.030	2005
Stivarga	$5.072	$1384.030	2012
Bexxar	$6.616	$1093.400	2003
Zelboraf	$7.144	$1047.950	2011
Spinraza	$1.604	$965.400	2016
Emtriva/Genvoya	$6.407	$951.000	2003
RTA-408	$71.746	$850.000	–
Diamyd	$5.799	$639.000	–
Zarnestra	$16.380	$628.000	–
ReoPro	$104.354	$625.000	1995
CMX001	$4.151	$613.500	–
Surfaxin	$38.388	$558.140	2012
Ixinity	$3.598	$508.300	2015
DTX301	$124.321	$481.733	–
Obizur	$7.014	$400.000	2014
haNK	$5.143	$350.460	–
Neuradiab	$313.768	$326.600	–
Increlex	$1.172	$326.270	2005
Treg	$1.804	$325.000	–
Prochymal	$4.959	$279.250	–
Amdoxovir	$19.124	$245.000	–
Horizant	$453.074	$219.990	2011
PA-457	$10.773	$218.830	–
TNFerade	$197.250	$205.900	–
Daytrana	$4.151	$200.000	2006
V2006	$11.215	$184.270	–
Gencaro	$2.377	$174.955	–
ThermoDox	$79.250	$170.000	–
SR9025	$36.127	$160.000	–
Rintega	$314.546	$145.100	–
GI-5005	$2.788	$122.600	–
Tolsura	$5.401	$96.700	2018
RiVax	$1.717	$93.000	–
Levovir	$41.201	$73.500	–
AEOL 10,150	$64.835	$69.460	–
Combipatch/Vivelle-dot	$4.150	$65.000	1998
Oncoprex	$404.693	$34.120	–
MBX-400	$10.934	$0.000	–
Total	$2,415.108	$50,671.236	
Total (approved only)	$670.208	$44,280.958	

Private funding excludes post-FDA approval funding

**Figure 2.**
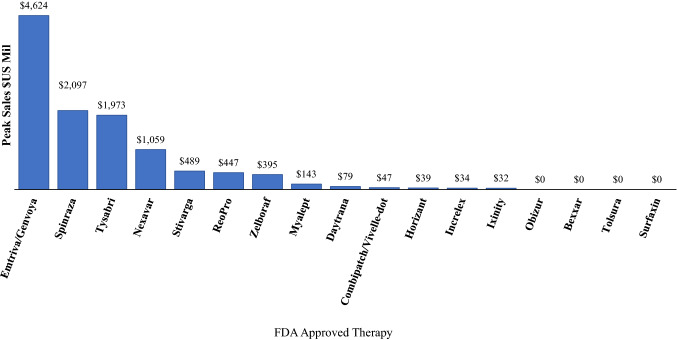
Peak Sales of FDA-Approved Therapies with at Least Three Years of Sales Data ($US Mil.).

Figure 2 shows peak sales for 17 of the 18 FDA-approved therapies having at least 3 years of post-approval sales data.^3^ Among these 17, only four had sales exceeding $1 billion annually while four had zero audited sales in the annual 10-K disclosure reports filed with the U.S. Securities and Exchange Commission.

**Figure 3.**
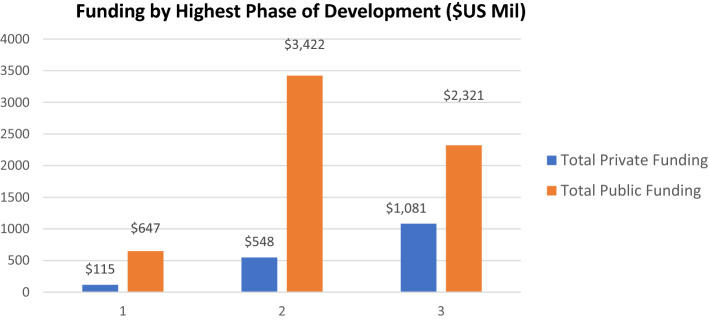
Funding by Highest Phase of Development Reached, for Projects not Resulting in an FDA-Approved Medicine ($US Million).

Figure 3 shows funding data for the 23 therapies in our cohort that did not receive FDA approval. As shown, the total private contribution toward those assets was four times that of the NIH, demonstrating that a large financial commitment is required from the private sector to determine the market potential of any new therapy, even when those products fail to gain FDA approval.

To conduct a formal statistical analysis of these data we estimate a logit model that relates the levels of public and private funding to the probability that a therapy receives FDA approval. For this analysis, the (natural) logarithm of each funding variable is used to minimize the potential impact of large funding values (i.e., outliers). One therapy (MDX-400) had zero private funding and was therefore excluded from the estimation sample since ln(0) is undefined.

Table 2 presents summary statistics and correlations for our model variables. The dependent variable “Approved” equals one if a therapy was FDA approved and zero otherwise. The mean of Approved (45%) indicates our sample is balanced in terms of approved versus not approved therapies. All correlations are significantly different from zero (*p* ≤ 0.05). Private funding and Approved are positively correlated, whereas public funding and Approved are negatively correlated. Public funding and private funding are negatively correlated meaning that, across all therapies, higher levels of public funding are associated with lower levels of private funding and vice-versa.

**Table 2:** Summary Statistics and Correlations.

Variable	Summary Statistics					Correlations	
	Mean	Median	SD	Min	Max	Approved	Private Funding
Approved	0.450	0.000	0.504	0	1	1.000	
Private Funding	19.766	19.604	1.291	17.345	23.838	0.489 (0.0014)	1.000
Public Funding	16.406	15.769	1.733	13.710	19.932	− 0.315 (0.0474)	− 0.335 (0.0346)

N = 40; P-value testing if correlation = 0 in parentheses. Funding variables measured as natural logarithm of their values

Tables 3 and 4 present the results of estimating the logit model. The results in Table 3 indicate overall model significance (Chi-square* p* ≤ 0.0015). The level of private funding is positive and significant (*p* < 0.02) for the likelihood of FDA approval, whereas the level of public funding negative but not significant (*p* < 0.22) at the conventional level of significance.

**Table 3:** Logistic Regression Results Predicting Probability of FDA Approval.

Variable	Estimate	SE	z-Stat	*P* value
Total Private Funding	1.0714	0.4366	2.4539	0.0141
Total Public Funding	− 0.2966	0.2438	− 1.2162	0.2239
Intercept	− 16.5187	9.6313	− 1.7151	0.0863
Log-likelihood	− 20.9998			
Chi-square	13.0515			
*P* value	0.0015			
Pseudo *R*-Square	0.237			
Observations	40			

Funding variables measured as natural logarithm of their values

**Table 4:** Average Partial Effect (APE) of a Change in Private and Public Funding on Probability of FDA Approval.

Variable	Estimate (APE)	Std. Error	z-stat	p-value
Total Private Funding	0.1890	0.0529	3.5708	0.0004
Total Public Funding	− 0.0523	0.0402	− 1.3027	0.1927

N = 40; Funding variables are measured as natural logarithm of their values

**Figure 4.**
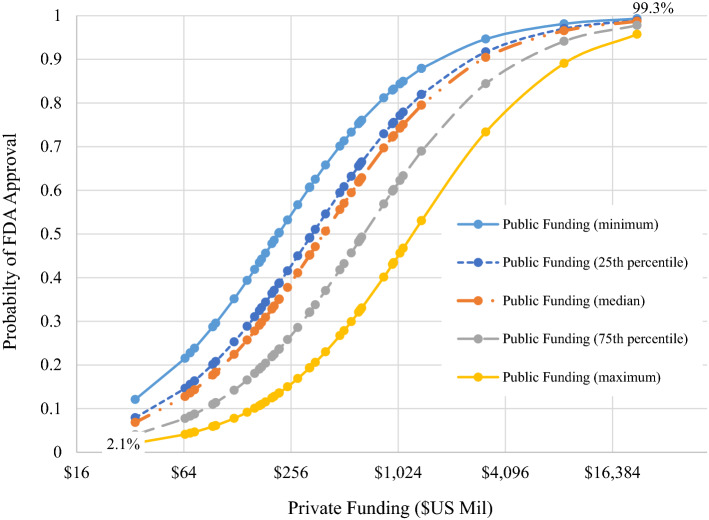
Estimated Relationship Between Total Private Funding and Probability of FDA Approval at Distinct Levels of Public Funding. Percentages shown in graph interior are the percent probability of approval when either (1) public funding is its sample maximum and private funding is its sample minimum (2.1%) or (2) public funding is its sample minimum and private funding is its sample maximum (99.3%).

To examine further these results, Fig. 4 depicts the estimated logit function over sample values of private funding at each of five different sample values of public funding (i.e., minimum, 25th percentile, median, 75th percentile, and maximum). Not unexpectedly, the entire logit function shifts down, and hence the probability of FDA approval at any level of private funding is lower, at higher levels of public funding.

Whereas the estimation results in Table 3 indicate overall model significance, the fact that the logit model is a nonlinear function of model coefficient means that the coefficient estimates in Table 3 do not indicate the true nature of the relationship between a given funding source and the probability of FDA approval. Instead, the impact of each funding variable on the probability of approval is given by its average partial effect (APE), which is the average of its marginal effect value at each sample observation.^4^

Table 4 presents the estimated APE for each funding variable. Since each variable is measured as the (natural) logarithm of its dollar value, each APE value indicates the change, on average, in the probability of FDA approval due to a doubling of the level of funding (equivalently, a 100% increase in the level of funding).^5^ As shown in Table 4, the relationship between private funding and the probability of FDA approval is positive and significant (*p* ≤ 0.0004). The relationship between public funding and the probability of FDA approval is negative but shows weak statistical significance (*p* < 0.193). By use of a one tailed test, greater confidence can be placed on the hypothesis that an increase in public funding decreases the probability of FDA approval (*p* ≤ 0.0965).^6^ Finally, comparing the absolute value of the estimated APE values, the impact of additional private funding on the probability of FDA approval is 3.6 ( =| 0.189/− 0.0523 |) times the impact of an increase in public funding.

**Figure 5.**
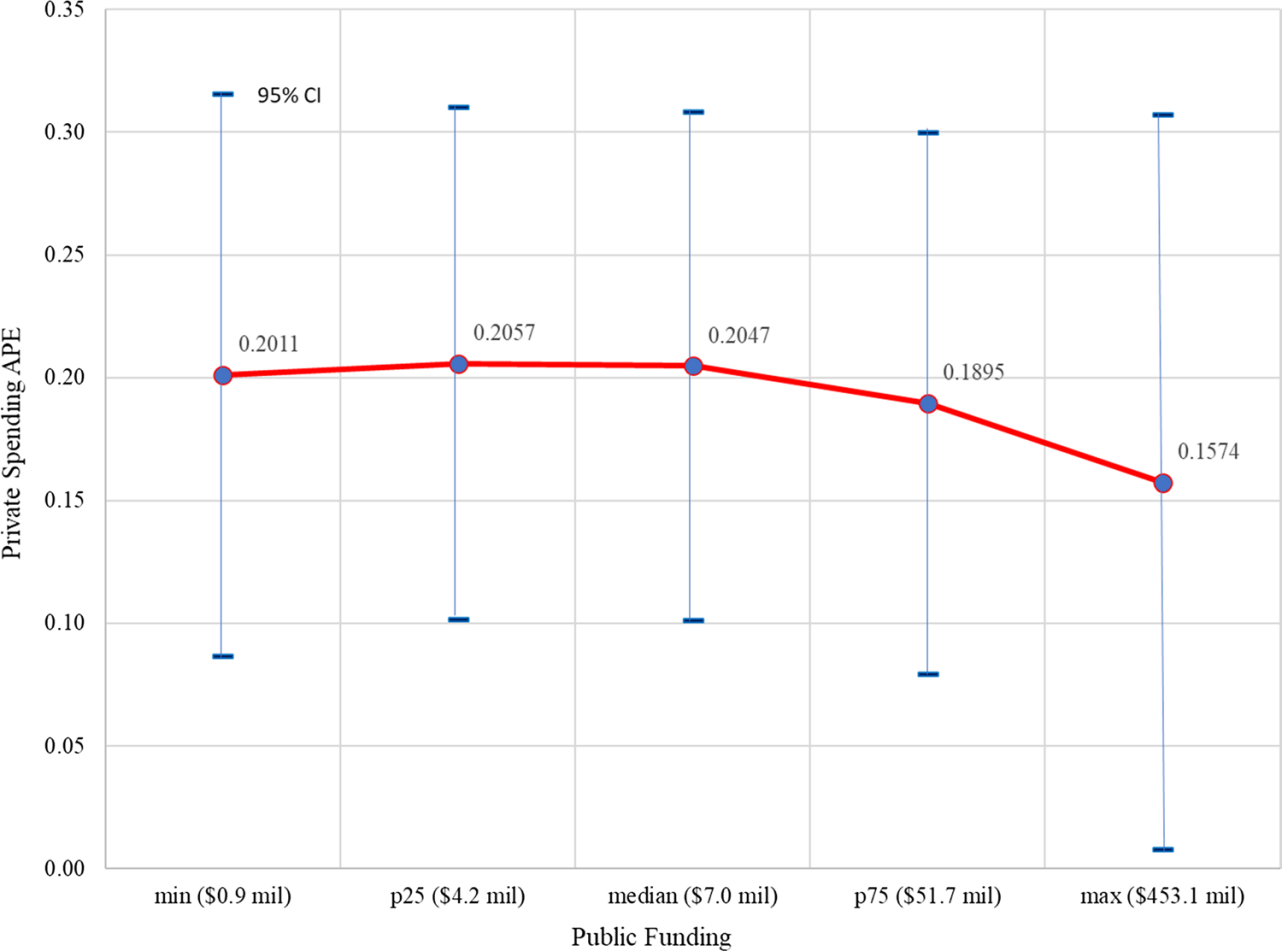
APE of Private Funding on Probability of FDA Approval over Values of Public Funding.

To further assess the effect of higher public funding, we can examine how the APE of private funding varies with the level of public funding. This relationship is the inherent “interaction effect” that arises from the nonlinearity of the logit function [15]. Figure 5 shows the values of the APE for private funding at different sample values of public funding (as done in Fig. 4). In Fig. 5, the value of the APE of private funding is essentially unchanged for values of public funding below its sample median, but the APE value then declines as the level of public funding rises above its sample median, suggesting a negative interaction effect. That is, the magnitude of the impact of higher private spending on the probability of FDA approval falls at higher levels of public funding. Testing that the value of the private funding APE at the minimum value of public funding exceeds its value at the maximum value of public funding, we can (weakly) reject the null hypothesis that the interaction effect is zero or positive (*p* ≤ 0.093).

## Discussion

Out study’s findings show no statistically significant relationship between the level of NIH funding and the probability that a therapy in clinical development will eventually receive FDA approval. In fact, there is evidence suggesting that the nature of this relationship may be negative (*p* ≤ 0.0965). In contrast, the relationship between private sector funding and the probability of FDA approval is positive and statistically significant (p ≤ 0.0004). Within our sample, when the level of public funding takes its maximum sample value and private funding takes is minimum sample value our model predicts the probability of FDA approval to be 2.1% Conversely, when public funding takes its minimum sample value and private funding takes its maximum sample value our model predicts the probability of FDA approval to be 97.3% When both public funding and private funding take their sample median value our model predicts the probability of FDA approval to be 49.6%.

The findings of our study are consistent with the view that NIH funding is primarily directed toward basic science research that establishes a base for the process of drug development. But importantly, our results indicate that, when NIH-funded research is linked to patented discoveries, additional public funding may have a significant (*p* ≤ 0.0965) negative impact on the probability of FDA approval. We conjecture that the estimated relationships between public and private funding and the probability of FDA approval may reflect a difference in the objective goals of each source of funding. Specifically, the extent of private funding is predicated on the expected return on the investments made, whereas the extent of public funding may instead be predicated on establishing only the viability of a given therapy, with only limited benefit–cost consideration given for the level or continuation of public funding. An as example, Horizant, the therapy with the highest percentage of public funding in our FDA-approved cohort, had nominal peak sales of only $39 million.

To address such issues, the NIH is increasingly turning to more market-oriented approaches of funding. Cooperative Research and Development Agreements (CRADAs) allow for licensing directly with U.S. companies, which led directly to the development of CAR-T therapies.^7^ As well, intermural “Z” grants allow for direct funding of specific researchers for the development of impactful biomedical innovation.^8^

Our study results demonstrate that basic discoveries require substantial additional investments, partnerships, and the shouldering of financial risks by the private sector if new therapies are to materialize as FDA-approved therapies. As a case in point, the majority of the funding for the 18 approved drugs in our study came from private sector investments; this was true across all stages of R&D and all therapeutic classes observed. It is important to note that the risk for private sector investment does not end at the moment the product is approved for commercialization. Most FDA-approved therapies had limited peak sales revenues, including four approved therapies that had zero revenues reported in company financial documents.

Lastly, it is essential to put these findings in the larger context of all drug development. Our study only researched therapies that were associated with NIH funding. However, medications typically draw on multiple patents critical to their development, and most of those patents are supported entirely through private investment. For example, for the 12 approved products in our analysis that appear in the FDA Orange Book, the NIH-supported patents we identified accounted for only 5 of the 41 total patents listed as protecting these products. Furthermore, a 2019 analysis of 197 top-selling therapies found that only 10.2% had at least one patent recorded in the Orange Book with a government interest statement or U.S. government agency assignee [7].

### Limitations

Ascertaining the relevance and importance of every NIH-supported patent identified within our search cohort was outside the scope of this analysis. The analysis also used public and commercially available business intelligence data sources to link NIH grants to products in the pipeline using patents listed in these sources as the link. This link is imperfect and appears to capture some NIH-supported patents that may be tangential or potentially even unrelated to the development of the pipeline products identified, thus overstating NIH investments and inventions related to these investigational therapies.

The analysis used publicly reported financial transaction data (e.g., equity, royalties, licensing, IPOs, acquisitions) as a proxy for industry investment. This does not explicitly include the considerable internal R&D investments typically made by biopharmaceutical firms after these funding transactions, so it is highly likely the total private contribution toward the commercialization of NIH created patents is understated.

Furthermore, while the extent to which the NIH RePORTER database underreports patents, the number of patents and products associated with NIH grants may also be understated. However, total NIH investment for identified products is not likely to be significantly affected, given our approach of including the total historical multi-year grant funding totals, even if patents were awarded before those grants were distributed in later years.

Due to the timeframe and focus of our research methodology, therapies developed via either CRADAs or intermural “Z” grants, some of which have since received FDA approval, are excluded from our cohort (cf. endnotes vii and viii).

Finally, while this study was able to further develop the original hypothesis of Munteanu [13], that spinning out from a publicly funded university was a negative indictor for FDA approval, our sample size (*N* = 40) potentially limited our ability to statistically validate, with a significance level below 5%, the hypothesis that NIH funding is a negative indicator of the probability of FDA approval. A larger cohort may be more likely to establish the statistical validity of that hypothesis.

## Conclusion

This paper addressed the issue of the relative contribution of the private and public (NIH) sector funding to the discovery and development of new therapies and the likelihood of FDA approval. While each sector makes important contributions, the findings of this study refute the false maxim that the public sector is solely responsible for all new innovations. This conclusion is consistent with the findings published in peer-reviewed scientific journals over the past several decades. Yet, many policymakers, journalists, and academicians continue to espouse the position that questions, or even denies, the role of the private sector in the discovery and development of innovative therapies. Such continued skepticism in the context of recent health policy debates around such issues as March-in Rights is a distraction for patients and their families desperately looking for curative therapies. It is hoped that this study’s objective examination of private versus public sector contributions to biopharmaceutical research and innovation will serve to better inform both current and future policy debates.

## Footnotes

1 These investments are made using not only direct funding but also a variety of other methods, including mergers and acquisitions, that are today an indispensable source of funds for, in particular, the U.S. biopharmaceutical ecosystem.

2 These were the National Cancer Institute, the National Heart Lung Blood Institute, the National Institute of Allergy and Infectious Diseases, the National Institute of Arthritis and Musculoskeletal and Skin Diseases, the National Institute of Diabetes and Digestive and Kidney Diseases, and the National Institute of Neurological Disorders and Stroke.

3 Peak sales is defined as the maximum value of annual sales across all years of reported sales. The medicine IMMU-132 (Trodelvy) is excluded from this analysis since it was only approved in 2020 and therefore had yet to achieve peak lifetime sales.

4 Since the logit function is nonlinear in model coefficients, the value of a variable’s marginal effect varies with the values of all model variables and hence is different at each sample observation. See e.g., [15].

5 For example, the APE value for private funding means that doubling private funding would increase the probability of FDA approval by 0.189, whereas the APE value for private funding indicates that a doubling of public sector funding would reduce the probability of approval by 0.0523.

6 This is the one-tail p-value when testing the null hypothesis that the APE of public funding is greater than or equal to zero against the alternative that the APE of public funding is negative.

7 Cooperative Research and Development Agreements (CRADAs) are designated under the Federal Technology Transfer Act of 1986 (P.L. 99–502) (which amended the Stevenson-Wydler Technology Innovation Act of 1980 (P.L. 96–480)). CRADAs are intended to speed the commercialization of technology, but also allow both parties to keep research results confidential for up to five years under the Freedom of Information Act. Given this, therapies developed via CRADAs were excluded from our research cohort as the potential lack of information could have biased our findings.

8 Therapies developed via intermural “Z” grants are excluded from this study since they were not disclosed by the NIH before the year 2007, which is after the base year (2000) of this study.
