# Correction: The Relative Contributions of NIH and Private Sector Funding to the Approval of New Biopharmaceuticals

**DOI:** 10.1007/s43441-022-00461-6

**Published:** 2022-09-16

**Authors:** Duane Schulthess, Harry P. Bowen, Robert Popovian, Daniel Gassull, Augustine Zhang, Joe Hammang

**Affiliations:** 1 Vital Transformation, Wezembeek-Oppem, Belgium; 2 McColl School of Business, Queens University of Charlotte, Charlotte, NC USA; 3 Global Healthy Living Foundation & Senior Health Policy Fellow Progressive Policy Institute, Washington, DC USA; 4 University of Southern California, Los Angeles, CA USA

## Correction to: Ther Innov Regul Sci (2022) 10.1007/s43441-022-00451-8

Analysis and results, paragraph 1 - the phrase “$2415 billion” should read “$2.415 billion”. The phrase “$50,671 billion” should read “$50.671 billion”. The phrase “$91,256 billion” should read “$91.256 billion”. Discussion Section, 1st paragraph, second to last sentence states: “Conversely, when public funding takes its minimum sample value and private funding takes its maximum sample value our model predicts the probability of FDA approval to be 97.3%” The value 97.3% should be 99.3%. Discussion section, 1st paragraph, 1st sentence begins: “Out study’s findings show no statistically significant relationship …”. “Out” should be “Our”. The dataset titles in Fig. 3 were reversed. The corrected Fig. 3 follows:

**Figure 3.**
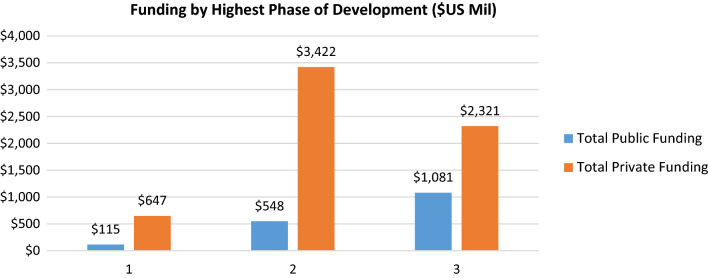
Funding by Highest Phase of Development Reached, for Projects not Resulting in an FDA-Approved Medicine ($US Million).

